# Interleukin 6 at menstruation promotes the proliferation and self-renewal of endometrial mesenchymal stromal/stem cells through the WNT/β-catenin signaling pathway

**DOI:** 10.3389/fimmu.2024.1378863

**Published:** 2024-05-03

**Authors:** Tianqi Li, Raymond H. W. Li, Ernest H. Y. Ng, William S. B. Yeung, Philip C. N. Chiu, Rachel W. S. Chan

**Affiliations:** ^1^ Department of Obstetrics and Gynaecology, School of Clinical Medicine, Li Ka Shing Faculty of Medicine, The University of Hong Kong, Hong Kong, Hong Kong SAR, China; ^2^ Centre for Translational Stem Cell Biology, The University of Hong Kong, Hong Kong, Hong Kong SAR, China; ^3^ Shenzhen Key Laboratory of Fertility Regulation, The University of Hong Kong Shenzhen Hospital, Shenzhen, China

**Keywords:** endometrial stem cells, stem cell niche, endometrial regeneration, WNT/β-catenin signaling, interleukin 6

## Abstract

**Background:**

At menstruation, the functional layer of the human endometrium sheds off due to the trigger of the release of inflammatory factors, including interleukin 6 (IL-6), as a result of a sharp decline in progesterone levels, leading to tissue breakdown and bleeding. The endometrial mesenchymal stem-like cells (CD140b^+^CD146^+^ eMSC) located in the basalis are responsible for the cyclical regeneration of the endometrium after menstruation. Endometrial cells from the menstruation phase have been proven to secrete a higher amount of IL-6 and further enhance the self-renewal and clonogenic activity of eMSC. However, the IL-6-responsive mechanism remains unknown. Thus, we hypothesized that IL-6 secreted from niche cells during menstruation regulates the proliferation and self-renewal of eMSC through the WNT/β-catenin signaling pathway.

**Methods:**

In this study, the content of IL-6 across the menstrual phases was first evaluated. Coexpression of stem cell markers (CD140b and CD146) with interleukin 6 receptor (IL-6R) was confirmed by immunofluorescent staining. *In vitro* functional assays were conducted to investigate the effect of IL-6 on the cell activities of eMSC, and the therapeutic role of these IL-6- and WNT5A-pretreated eMSC on the repair of injured endometrium was observed using an established mouse model.

**Results:**

The endometrial cells secrete a high amount of IL-6 under hypoxic conditions, which mimic the physiological microenvironment in the menstruation phase. Also, the expression of IL-6 receptors was confirmed in our eMSC, indicating their capacity to respond to IL-6 in the microenvironment. Exogenous IL-6 can significantly enhance the self-renewal, proliferation, and migrating capacity of eMSC. Activation of the WNT/β-catenin signaling pathway was observed upon IL-6 treatment, while suppression of the WNT/β-catenin signaling impaired the stimulatory role of IL-6 on eMSC activities. IL-6- and WNT5A-pretreated eMSC showed better performance during the regeneration of the injured mouse endometrium.

**Conclusion:**

We demonstrate that the high level of IL-6 produced by endometrial cells at menstruation can induce the stem cells in the human endometrium to proliferate and migrate through the activation of the WNT/β-catenin pathway. Treatment of eMSC with IL-6 and WNT5A might enhance their therapeutic potential in the regeneration of injured endometrium.

## Introduction

The human endometrium undergoes dynamic shedding and regeneration in a menstrual cycle in response to ovarian hormones (1, 2). In the secretory phase, the stromal compartment of progesterone-primed endometrium will decidualize for the preparation of embryo implantation (3). In the absence of pregnancy, progesterone levels dramatically decline, leading to concatenate physiological changes in the endometrium such as tissue edema, increased vessel permeability, and the recruitment of various leukocytes, thus initiating menstruation (4–6). Therefore, menstruation was hypothesized to be an inflammatory event (7). During this period, the induction of inflammatory factors, including interleukin 6 (IL-6), was documented, and angiorrhexis further stimulated the secretion of cytokines by exposing endometrial compartments to a physiological hypoxic condition (8–10). On the other hand, an influx of inflammatory cells into the wounded endometrium, together with the chemokines in the local environment, produce a series of cytokines and mediators that accelerate thrombosis, angiogenesis, and re-epithelialization, indicating their role in endometrial repair (11).

Increasing evidence shows that the rapid regeneration of the endometrium after menstruation is mainly due to the existence of endometrial mesenchymal stem cells (eMSC) (12). The eMSC was identified in upper functionalis as well as basalis near the endometrial–myometrial junction and has coexpression of two perivascular markers CD140b and CD146 (13). Transcriptomic analysis of eMSC revealed their expression of inflammation-responsive genes, and *in vivo* studies demonstrated their immunoregulatory roles during the regenerating processes of injured mouse endometrium (14, 15). Previously, our group reported that the endometrial epithelial and stromal cells from the menstruation phase acted as niche components by enhancing the self-renewal and clonogenic activity of eMSC (16). It is also demonstrated that the WNT/β-catenin signaling pathway is involved in the communication of niche cells from the menstrual phase and our eMSC. Since the stimulating effect was only observed in niche cells from the menstruation phase but not from the proliferative phase, we hypothesized the presence of a unique microenvironment that activates the eMSC during endometrial shedding. From a panel of cytokines that regulate endometrial regeneration and maintain MSC “stemness”, IL-6 has been shown to induce the proliferation of eMSC and elevate phenotypic expression (17, 18). In this study, we hypothesized that IL-6, a natural factor in the endometrium, is required for the facilitation of endometrial regeneration at the time of menstruation. Our finding shows that the IL-6 secreted from niche cells during menstruation regulates the proliferation and self-renewal of eMSC through the WNT/β-catenin signaling pathway.

## Materials and methods

### Human endometrial sample collection

Full thickness of human endometrial tissue (*n* = 27, **Supplementary Table S1**) was collected from women who were aged between 41 and 50 years, had regular menstrual cycles, and underwent hysterectomy for benign pathologies. Those with a history of exogenous hormonal therapy within 3 months before the surgery were excluded. The menstrual phase of each sample was defined by an experienced histopathologist into proliferative (*n* = 17) and secretory (*n* = 10) phases based on the hematoxylin and eosin-stained tissue sections. Endometrial aspirations were obtained from six women with regular menstrual cycles aged from 32 to 40 years (**Supplementary Table S2**) who attended the infertility clinic on days 2–3 of their menstrual cycle for assisted reproduction. Written consent was acquired from participants prior to their recruitment to this study, and ethical approval was obtained from the Institutional Review Board of The University of Hong Kong/Hospital Authority Hong Kong West Cluster (UW20-465) and the Institutional Review Board of the University of Hong Kong Shenzhen Hospital ( [2018]94).

### Isolation of endometrial cells

The isolation procedures for full-thickness endometrial tissue followed the protocol of our previous studies (19, 20). Briefly, endometrial tissue was minced and digested with phosphate-buffered saline (PBS) containing deoxyribonuclease type I (40 μg/ml, Worthington Biochemical Corporation, Freehold, NJ, USA) and collagenase III (0.3 mg/ml, Worthington Biochemical Corporation) at 37° for 1 h. After two rounds of digestion, the cell suspension was filtered through 40-μm sieves (BD Bioscience, San Jose, CA, USA), and dispersed cells were acquired. Red blood cells and leukocytes were excluded by Ficoll–Paque (GE Healthcare, Uppsala, Sweden) and anti-CD45 antibody-coated Dynabeads (Invitrogen, Waltham, MA, USA), respectively. Anti-CD326 antibody-coated microbeads (Miltenyi Biotec Inc., San Diego, CA, USA) were applied to separate epithelial cells from the stromal population. Freshly isolated epithelial cells are seeded onto 10-cm dishes (BD Biosciences, San Jose, CA, USA) coated with 0.2% gelatin, while stromal cells will be seeded onto 10-cm dishes coated with fibronectin (1 mg/ml, Invitrogen). Both epithelial or stromal cells are cultured in a growth medium with 10% FBS (Invitrogen), 1% penicillin (Invitrogen), and 1% l-glutamine (Invitrogen) in DMEM-F12 (Sigma-Aldrich, St Louis, MA, USA) in a humidified carbon dioxide incubator at 37° until reaching 80% confluence. The medium was changed every 7 days.

### Magnetic selection of endometrial mesenchymal stem-like cells

Two positive selections using magnetic microbeads were used for the acquisition of CD140b^+^CD146^+^ eMSC (19). Firstly, *in vitro*-expanded stromal cells were incubated with PE-conjugated anti-CD140b antibody (R&D Systems, Minneapolis, MN, USA) for 45 min at 4° and then with antimouse IgG1-coated magnetic microbeads (Miltenyi Biotec Inc.) for another 15 min. After applying the cell suspension to MS columns (Miltenyi Biotec Inc.) in the magnetic field, CD140b^+^ cells were obtained and further cultured for 7 to 10 days to allow microbead degradation during cell expansion. For the second round of selection, cells were incubated with anti-CD146 antibody-coated microbeads (Miltenyi Biotec Inc.) for 15 min at 4°. CD140b^+^CD146^+^ eMSC were then obtained from column separation. Stromal cells at passages 1–3 were employed in this study.

### Cell proliferation assay

The proliferative activity of eMSC was determined using the CyQUANT™ Cell Proliferation Kit (Thermo Fisher Scientific, Waltham, MA, USA). According to the manufacturer’s instructions, eMSC were seeded at a density of 1,000 cells per well into a 96-well plate and cultured in different treatment conditions. Cells cultured in a growth medium served as control. After 3 days of culture, the cells were washed with PBS, followed by a 1-h incubation with 100 μl of dye-binding solution in the dark at 37°. Fluorescence intensity was measured using a fluorescence microplate reader with an excitation wavelength of 485 nm and an emission wavelength of 520 nm.

### *In vitro* colony-forming assay

To examine the colony-forming capacity, freshly isolated eMSC were seeded at a density of 30 cells/cm^2^ under different treatment conditions. After the 2-week coculture, colonies were fixed in 10% formalin, washed with H_2_O, and stained with Toluidine Blue (1 mg/ml, Sigma-Aldrich) for 5 min. Cell clumps containing more than 500 cells were considered colonies. The total number of colonies formed in each group was recorded, and the clonogenic efficiency was calculated as the number of colonies formed divided by the number of cells seeded multiplied by 100% (20).

### Flow cytometry

The multicolor flow cytometry technique was applied to examine the phenotypic expression of eMSC and markers under different treatment conditions after the 2-week culture. Harvested cells were double stained with PE-conjugated anti-CD140b antibody (2.5 µg/ml, PR7212 clone, mouse IgG1, R&D Systems) and FITC-conjugated CD146 (5 µg/ml, OJ79c clone, mouse IgG1, Thermo Fisher Scientific) in 0.5% BSA/PBS at 4° in the dark for 45 min. The Fluorescent Minus One (FMO) control was included for each antibody. The fluorescence of labeled cells was detected with CytoFlex™ flow cytometer (Beckman Coulter, CA, USA), and the obtained data were analyzed with FlowJo software (Tree Star Inc., Ashland, OR, USA) (21). Triple staining of APC-conjugated anti-IL-6R (5 µg/ml, UV4 clone, mouse IgG, BioLegend, San Diego, CA, USA) together with stem cell markers (CD140b and CD146) was performed using endometrial stromal population. The gating strategy is shown in **Supplementary Figure S1**.

### RNA extraction, reverse transcription-quantitative polymerase chain reaction

Total RNA extraction was performed using Absolutely RNA Microprep Kit (Agilent Technologies, La Jolla, CA, USA) following the manufacturer’s instructions. The Nanodrop 2000 Spectrophotometer (Thermo Fisher Scientific) was used to measure the RNA concentration. After normalization, reverse transcription from RNA to cDNA was accomplished by the Prime Script RT Reagent Kit (Takara Bio Inc., Japan, San Jose, CA, USA). Reverse transcription-quantitative polymerase chain reaction (qPCR) was then conducted for measurement of target gene expression (*IL-6*, Hs00174131_m1; *CXCR4*, Hs00607978_s1; Thermo Fisher Scientific). The detection was done by using the 7500 Real-Time PCR system (Applied Biosystems, Waltham, MA, USA). Gene expression was presented as relative mRNA expression using the 2^−ΔΔCt^ method and 18S served as an internal control of each sample.

### Immunofluorescence staining

For IF staining of cells, around 8,000–10,000 cells were transferred onto each 3-aminopropylterithoxy saline (AAS, Sigma-Aldrich)-coated slide using the Shandon Cytospin Centrifuge (Thermos Electron, USA, Cambridge, UK) at the speed of 1,700 rpm for 10 min. Cell fixation was accomplished by 4% paraformaldehyde (PFA) for another 10 min at room temperature. For tissues, paraffin-embedded endometrial tissues were attached to the AAS-coated slides, followed by dewax, antigen retrieval, denaturation, and quenching procedures. To visualize protein expression in the nucleus, cells were permeabilized with 0.1% Triton X-100 for 15 min. Primary antibodies (**Supplementary Table S3**) were added and incubated overnight at 4°. On the following day, corresponding secondary antibodies were then added at room temperature for 1 h. Cell nuclei were stained with DAPI (Thermo Fisher Scientific), and the slides were mounted with a fluorescence mounting medium (Dako, Glostrup, Denmark). The fluorescent images were captured by a Carl Zeiss LSM 780 inverted confocal microscope and analyzed by Zeiss LSM Zen 2010 software (Carl Zeiss, Munich, Germany) at the Centre for PanorOmic Sciences (CPOS), the University of Hong Kong.

### Western blotting

Protein lysate was acquired by incubating cultured eMSC in cell lysis buffer (Ambion, Grand Island, NY, USA) containing proteinase inhibitor (PI, Calbiochem., Millipore, Burlington, MA, USA) and phosphatase inhibitor (PhI, Calbiochem., Millipore). In total, 30 µg of denatured protein was separated in 10% SDS-PAGE gel and transferred onto PVDF membranes (Immobilon™-P, Millipore). After blocking with 5% milk, the membranes were incubated with rabbit polyclonal Active β-Catenin (1:1,000, Cell Signaling Technology, Danvers, MA, USA) or mouse monoclonal β-actin (1:2,000, Sigma-Aldrich) overnight at 4°. The secondary antibodies used for detection were horseradish peroxidase-conjugated antirabbit IgG (1:5,000, GE Healthcare) and antimouse IgG (1:5,000, GE Healthcare), respectively. Protein bands were visualized by the Enhanced Chemiluminescence (ECL) Kit (Advansta, San Jose, CA, USA). ImageJ software was used in data analysis, and the calculation of target protein expression was normalized against the housekeeping protein β-actin.

### Luciferase assay

To investigate the role of IL-6 on WNT/β-catenin activities, a luciferase assay was conducted. The eMSC were cotransfected with 4 μg of TOPflash/Fopflash vector and 1 μg of pRL-TK (Renilla-TK-Luciferase vector, Promega, Madison, WI, USA) using Lipofectamine 3000 (Invitrogen). After 18 h of transfection, the medium was changed to growth medium, and some cells were treated with human recombinant IL-6 protein (500 pg/ml or 1,000 pg/ml; Pepro Tech, USA, London, England) or human recombinant WNT3A as positive control (0.01 μg/ml, R&D Systems) for 48 h. After, the luciferase activity was measured using a GLOMAX™ 96 microplate luminometer, and the TOP/FOP ratio was calculated for comparison.

### Cell migration assay

The migrating capacity of eMSC with different treatment conditions was evaluated using the QCM™ 24-Well Fluorimetric Cell Migration Kit (EMD Millipore Corp., Burlington, MA, USA). One day before the experiment, the FBS-free medium was supplemented to the cells in replacement of the growth medium; 3 × 10^4^ eMSC were seeded into each hanging insert for a 24-well plate. The medium was replaced with different concentrations of human recombinant IL-6 or WNT5A protein. After 72 h of culture, cell detachment buffer (EMD Millipore) was added to the plate and incubated with shaking for 30 min at 37°, Followed by adding the reaction buffer comprising CyQUANT GR Dye (EMD Millipore) and cell lysis buffer (EMD Millipore) and incubating on a shaker for another 15 min at room temperature. The degree of cell migration was indicated by the fluorescence intensity and detected using a fluorescence microplate reader with an excitation wavelength of 485 nm and an emission wavelength of 535 nm.

### Collection of conditioned medium for ELISA

Endometrial stromal or epithelial cells were seeded at a density of 1 × 10^4^ cells/well onto 24-well plates. Unattached cells after a 24-h cell culture were discarded, and fresh growth medium was then supplemented. For the normoxic culture condition, cells were incubated in a humidified cell incubator with 21% O_2_. For hypoxic cultures, culture plates were placed into an environment filled with the gas mixture of 2% O_2_, 5% CO_2_, and 93% N_2_ (*v/v*) at 37°C in a sealed chamber (Thermo Fisher Scientific). Conditioned medium was collected after 2 days’ culture.

### Enzyme-linked immunosorbent assay

The concentration of IL-6 in a conditioned medium was measured using an interleukin 6 human uncoated enzyme-linked immunosorbent assay (ELISA) kit (Thermo Fisher Scientific). As instructed by the manufacturer, 96-well plates were coated with antihuman IL-6 capture antibody overnight at 4°. On the following day, the plates were washed and blocked with the assay buffer for 1 h at room temperature. A standard curve was made by twofold serial dilutions of recombinant IL-6 protein at concentrations ranging from 7.8 to 1,000 pg/ml. Standards and samples were then added to the prepared wells and incubated for another overnight at 4°. The next day, the detection antibody was added into each well after proper washing steps and incubated for 1 h at room temperature. Subsequently, the streptavidin-HRP solution was applied to the wells for another 30 min. Five to seven rounds of washing steps were carried out before adding the 3,3′,5,5′-tetramethylbenzidine (TMB, BD OptiEIA™) substrate for a 15-min incubation, and the reaction was terminated with Stop Solution 2N H_2_SO_4_. Absorbance was read at a wavelength of 450 nm by a spectrometer (Beckman, USA), and values at 570 nm were subtracted for analysis.

### Animal and housing conditions

In this study, 6-week-old NOD-SCID female mice were used to establish the endometrial injury model. The mice were purchased and kept in the Centre of Comparative Medicine Research at the University of Hong Kong. All experimental procedures performed were approved by the Committee on Use of Live Animals in Teaching and Research, the University of Hong Kong. The mice were housed under standard laboratory conditions with a 12-h light/12-h dark cycle. A maximum of six mice were housed in one cage. Water and food are freely accessed.

### Animal study design

Electrocoagulation was used for the induction of endometrial injury, as reported previously (15). Briefly, after anesthetization by intraperitoneal injecting ketamine (10 g/kg) and xylazine (80–100 mg/kg), a vertical incision was made in the abdominal wall of the mice at diestrus for exposure of the uterine horn. The monopolar electrode was inserted into the lumen through a small incision made in the upper region of the right uterine horn. Electrocoagulation was performed with 50 W power for 3–4 s. While electrifying, the electrode was gradually moved out at a constant pace to ensure the damage to the entire endometrial surface. After returning the uterine horn to the abdominal cavity, the abdominal wall and skin layer were sutured and disinfected. The left uterine horn was untouched as a control.

To investigate the *in vivo* effect of IL-6 and WNT5A on the activities of eMSC, a total of 32 NOD-SCID female mice were randomly allocated into one of eight experimental groups (**Table 1**). In pretreatment groups, eMSC were cultured with the respective recombinant protein(s) for a week before the date of transplantation. For the eMSC transplantation group, 5 × 10^5^ eMSC labeled with CM-Dil fluorescent dye were injected into each uterine horn. The mice were euthanized, and the uterine horns were collected at postoperative day 5 (*n* = 4/group). Part of the harvested uterine horns were fixed in 4% paraformaldehyde overnight and processed into paraffin blocks for histological analysis. The remaining part was dissected for gene expression analysis.

**Table 1:** Animal experimental groups for endometrial injury.

	Nontransplantation groups	eMSC-transplantation groups
Nontreated	20 μl of PBS	Nontreated eMSC in 20 μl of PBS
IL-6	PBS + 1,000 pg/ml rhIL-6	eMSC pretreated with 1,000 pg/ml rhIL-6 in rhIL-6-supplemented PBS
WNT5A	PBS + 0.01 μg/ml rhWNT5A	eMSC pretreated with 0.01 μg/ml rhWNT5A in rhWNT5A-supplemented PBS
IL-6 + WNT5A	PBS + 500 pg/ml rhIL-6 + 0.005 μg/ml rhWNT5A	eMSC pretreated with 500 pg/ml rhIL-6 and 0.005 μg/ml rhWNT5A in IL-6/rhWNT5A-supplemented PBS

### Evaluation of endometrial thickness

After being stained with hematoxylin and eosin, endometrial sections were examined under a Zeiss microscope (Carl Zeiss), and images were captured using TCapture software. Ten consecutive transverse sections from a single uterine horn were assessed. The outer and inner regions of the transverse sections were manually drawn using the Image Pro Plus software (version 6.0; Media Cybernetics Inc., Rockville, MD, USA). The distance in between was measured to calculate the average diameter of the endometrial cavity. The injured uterine horn (right) was normalized against the control uterine horn (left) from the same animal.

### Statistical analysis

Data analysis was accomplished by using the GraphPad PRISM software (version 8.0; GraphPad Software Inc., San Diego, CA, USA) and tested for normal distribution using the Shapiro–Wilk test. Statistical differences between the two groups were determined by a two-tailed unpaired Student’s *t*-test for parametric data and Mann–Whitney *U* test for nonparametric data. For comparison among multiple groups, one-way ANOVA followed by Tukey’s test was used for parametric data, and Kruskal–Wallis test followed by Dunn’s *post-hoc* test was used for nonparametric data. Data are presented as mean ± standard deviation (SD). *p* < 0.05 was considered statistically significant.

## Results

### IL-6 and IL-6R were present in endometrial physiological conditions

**Figure 1:**
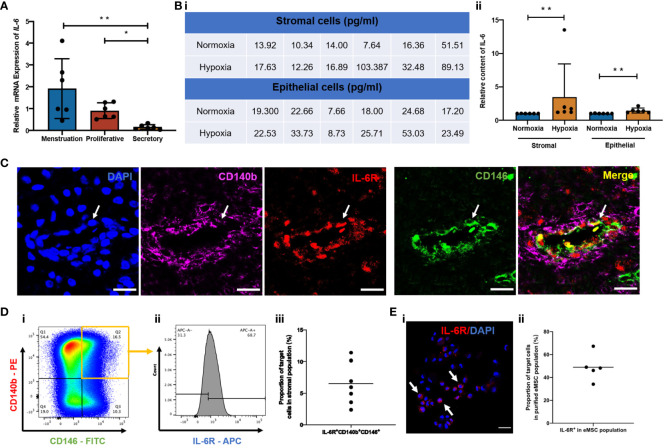
Expression of IL-6 across the menstrual cycle and presence of IL-6R in the human endometrium. **(A)** Relative mRNA expression of IL-6 in stromal cells at different phases of the menstrual cycle normalized to the proliferative phase (*n* = 6). **(B)** (i) Quantification values and (ii) of relative secretion of IL-6 cytokine from stromal or epithelial cells under normoxia (blue bars) or hypoxia (yellow bars). Normalized to hypoxia (*n* = 6). **(C)** Representative immunofluorescence images showing the coexpression of CD140b (pink), CD146 (green), and IL-6R (red) in human endometrium; scale bar: 20 μm. **(D)** (i, ii) Representative FCM results and (iii) proportion of IL-6R^+^CD140b^+^CD146^+^ cells in the stromal population (*n* = 7). **(E)** (i) Representative immunofluorescent image showing the expression of IL-6R (red) (white arrows) and (ii) the proportion of IL-6R^+^ cells in purified CD140b^+^CD146^+^ population (*n* = 5). Scale bar: 50 μm. Results are presented as mean ± SD; ^*^ *p* < 0.05; ^**^ *p* < 0.01. IL-6, interleukin 6; eMSC, endometrial mesenchymal stem cells.

To validate the hypothesis that the capacity of endometrial cells to secrete IL-6 varies across the menstrual cycle, gene expression of IL-6 in freshly isolated stromal cells from different menstrual phases was assessed using qPCR. The IL-6 expression was significantly higher during menstruation and the proliferative phase when compared to the secretory phase (*p* < 0.01, *n* = 6, **Figure 1A**). Since menstruation exposes the endometrial cells to a hypoxic condition, we further evaluated the secretion level of IL-6 from endometrial stromal and epithelial cells cultured under different oxygen levels (21% O_2_ and 2% O_2_) for 48 h. The endometrial cells under hypoxia secreted more IL-6 than those under normoxia (*p* < 0.01, *n* = 6, **Figure 1B**).

Since IL-6 triggers biological functions by binding to the IL-6 receptor (IL-6R) (22), we further checked the presence of IL-6R in human endometrium and, specifically, in our eMSC population. By immunofluorescent staining, we confirmed the expression of IL-6R in human endometrial tissue. The fluorescence signals of IL-6R and eMSC markers CD140b and CD146 (white arrow, **Figure 1C**) were localized to the perivascular region of the endometrium—a physiological location for the putative endometrial stem cells (13). Flow cytometry analysis revealed that around 6.5% of total endometrial stromal cells were IL-6R^+^CD140b^+^CD146^+^ (*n* = 7; **Figure 1D**). In histological sections, some of the CD140b^+^CD146^+^ cells coexpressed IL-6R (white arrow, **Figure 1C**), suggesting our eMSC could respond to IL-6 in the microenvironment. It is worth noting that not all eMSC expressed IL-6R; only 49.9% of the cells were IL-6R^+^ (*n* = 5, **Figure 1E**).

### IL-6 facilitated the proliferation and self-renewal of eMSC

**Figure 2:**
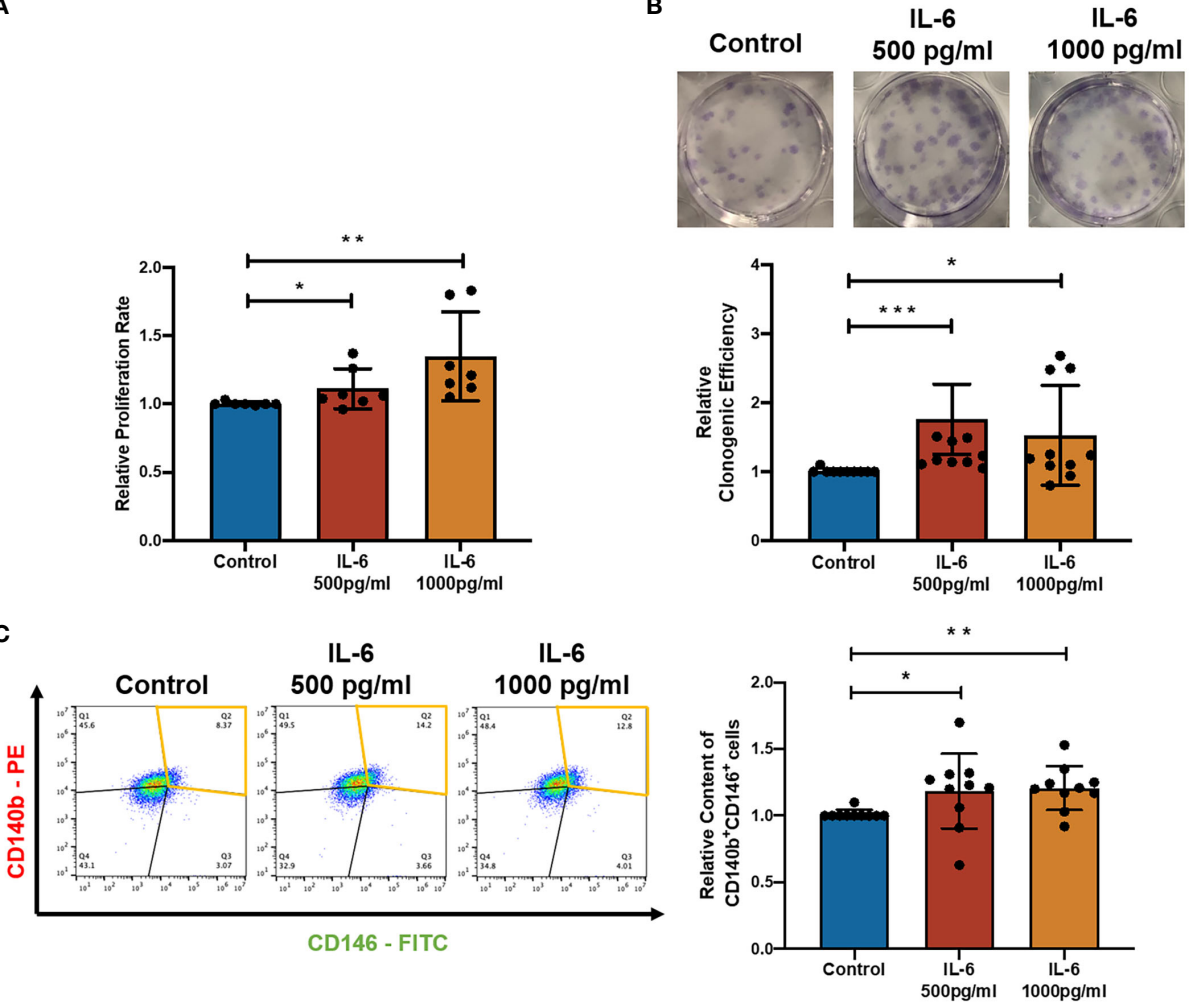
Effect of recombinant IL-6 protein on eMSC activities. **(A)** The relative proliferation rate of eMSC after treatment with GM (blue) and the addition of rhIL-6 at 500 pg/ml (red bar) and 1,000 pg/ml (yellow bar) (*n* = 8). **(B)** Representative images showing the distribution of colonies and the relative clonogenic efficiency of eMSC colonies (*n* = 10). **(C)** Representative images and relative proportion of CD140b^+^CD146^+^ cells (*n* = 10). Results are presented as mean ± SD; ^*^ *p* < 0.05; ^**^ *p* < 0.01; ^***^ *p* < 0.001. IL-6, interleukin 6; GM, growth medium; eMSC, endometrial mesenchymal stem cells.

To characterize the effect of IL-6 on eMSC activities, IL-6 at concentrations found in the menstrual endometrial cells was used in the eMSC coculture system (16). Treatment with recombinant IL-6 protein significantly induced the proliferation of eMSC (**Figure 2A**, *n* = 8, *p* < 0.05 for 500 pg/ml, *p* < 0.01 for 1,000 pg/ml). When eMSC were seeded at a low density (30 cells/cm^2^), the addition of IL-6 during the 2-week cell culture efficiently increased the clonogenic efficiency (**Figure 2B**, *n* = 10, *p <* 0.001 for 500 pg/ml, *p* < 0.05 for 1,000 pg/ml) and the relative proportion of cells coexpressing CD140b and CD146 (**Figure 2C**, *n* = 10, *p* < 0.05 for 500 pg/ml, *p* < 0.01 for 1,000 pg/ml) when compared to eMSC cultured in growth medium.

### WNT/β-catenin signaling pathway was involved during the regulation of IL-6 on eMSC

**Figure 3:**
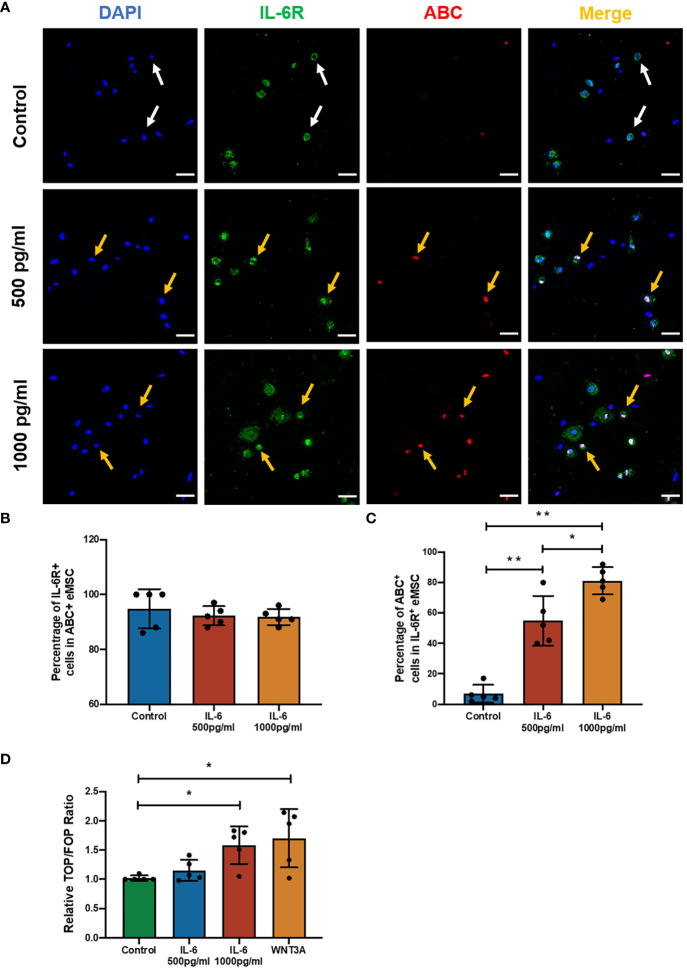
The effect of IL-6 on the WNT/β-catenin signaling pathway. **(A)** Representative immunofluorescence images showing the expression of ABC (red) and IL-6R (green) in eMSC after treatment with or without different concentrations of rhIL-6 protein; scale bar: 50 μm. **(B)** The relative percentage of IL-6R^+^ cells in the ABC^+^ eMSC population in GM (blue bar) or addition with rhIL-6 at 500 pg/ml (red bar) and 1,000 pg/ml (yellow bar) (*n* = 5). **(C)** Relative percentage of ABC^+^ cells in IL-6R^+^ eMSC (*n* = 5). **(D)** The activity of the Wnt/β-catenin signaling pathway was determined by the relative TOP/FOP ratio of eMSC in GM (green bar) or addition with different concentrations of rhIL-6 (blue and red bars) and 0.01 μg/ml rhWNT3A protein (yellow bar, positive control) (*n* = 5). Results are presented as mean ± SD; ^*^ *p* < 0.05; ^**^ *p* < 0.01. IL-6, interleukin 6; GM, growth medium; ABC, active β-catenin; eMSC, endometrial mesenchymal stem cells.

Previously, it was reported that coculture with the menstrual phase endometrial epithelial and stromal cells could significantly increase the level of active β-catenin (ABC) in eMSC-derived colonies (16). The ELISA result showed a five- to sixfold increase in IL-6 level in the spent medium after coculture with menstrual phase endometrial cells when compared with eMSC monoculture. These observations indicated the potential involvement of the WNT/β-catenin signaling pathway during IL-6 regulation of the eMSC population. As expected, the presence of IL-6 in the growth medium induced the expression of ABC in eMSC, which functions as an indicator of the activation of WNT/β-catenin signaling (**Figure 3A**). Among all treatment groups, the majority of the ABC^+^ eMSC possessed the expression of IL-6R, and the proportion of IL-6R^+^ individuals was similar (control: 94.8% ± 7.2%; 500 pg/ml: 92.2% ± 3.5%; 1,000 pg/ml: 91.8% ± 3.0%, *n* = 5, **Figure 3B**), indicating that a combination of exogenous IL-6 and presence of IL-6R on eMSC activated the WNT/β-catenin signaling. The percentage of ABC^+^ cells in the IL-6R^+^ eMSC displayed a dose-dependent increase with the concentration of IL-6 added. In the control group, only a limited number of IL-6R^+^ eMSC (6.8% ± 5.9%) expressed ABC, which was significantly less than the IL-6-treated groups (*p* < 0.01). Treatment with IL-6 significantly elevated the proportion of ABC^+^ cells in the IL-6R^+^ eMSC from 55.0% ± 16.3% to 81.2% ± 8.9% when the concentration of IL-6 changed from 500 to 1,000 pg/ml (*n* = 6, *p* < 0.05, **Figure 3C**). In addition, the TCF/LEF luciferase activities increased in the group treated with 1,000 pg/ml of IL-6 (*n* = 5, *p* < 0.05, **Figure 3D**). Based on this finding, 1,000 pg/ml of IL-6 was used for subsequent experiments.

**Figure 4:**
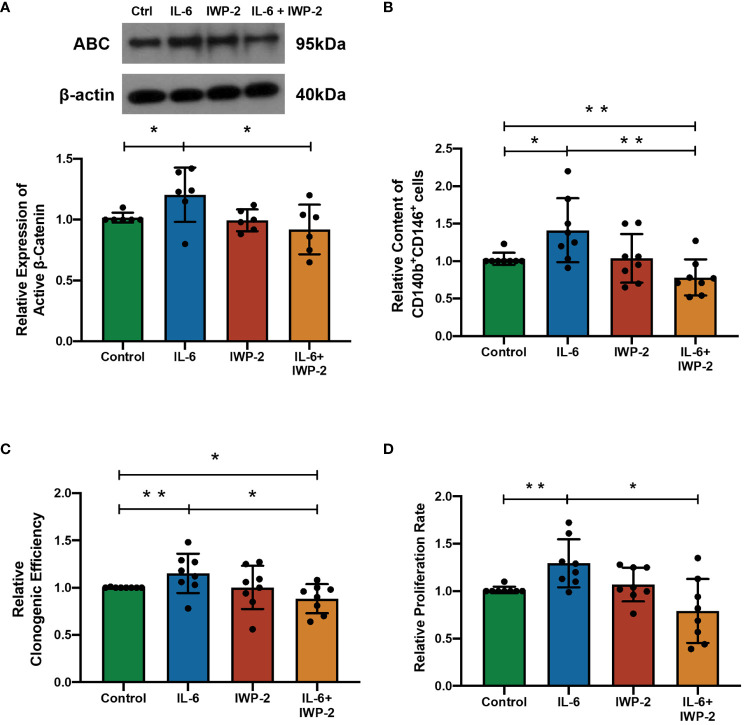
The effect of IL-6 on eMSC activities after inhibition of WNT/β-catenin processing with IWP-2. **(A)** The protein levels of ABC in eMSC in GM (green bar), 1,000 pg/ml of rhIL-6 (blue bar), IWP-2 at 1.25 μM (red bar), and 1,000 pg/ml of rhIL-6 with IWP-2 at 1.25 μM (yellow bar). Representative Western blotting bands of ABC (*n* = 6, normalized to β-actin). **(B)** Relative proportion of CD140b^+^CD146^+^ cells (*n* = 8). **(C)** Relative clonogenic efficiency of eMSC colonies (*n* = 8). **(D)** The relative proliferation rate of eMSC (*n* = 8). Results are presented as mean ± SD; ^*^ *p* < 0.05; ^**^ *p* < 0.01. IL-6, interleukin 6; GM, growth medium; ABC, active β-catenin; eMSC, endometrial mesenchymal stem cells.

In order to determine the importance of IL-6 for the observed effects, the impact of blocking WNT processing and secretion with IWP-2 was studied. IL-6 elevated the ABC expression (*n* = 6, *p* < 0.05, **Figure 4A**), the phenotypic expression (*n* = 8, *p* < 0.01, **Figure 4B**), clonogenicity (*n* = 6, *p* < 0.05, **Figure 4C**), and proliferation (*n* = 6, *p* < 0.05, **Figure 4D**) of eMSC. These stimulatory effects were abolished after the addition of IWP-2 to the culture system (ABC: *p* < 0.05, **Figure 4A**; phenotypic expression: *p* < 0.01, **Figure 4B**; clonogenic efficiency: *n* = 8, *p* < 0.05, **Figure 4C**; proliferation: *n* = 6, *p* < 0.05, **Figure 4D**). No differences were observed between the control group and the IWP-2 group in all the assays performed. Taken together, these findings provided evidence that the WNT/β-catenin signaling mediated the regulation of IL-6 on eMSC activities.

### The synergistic effect of IL-6 and WNT5A did not exist during eMSC regulation

**Figure 5:**
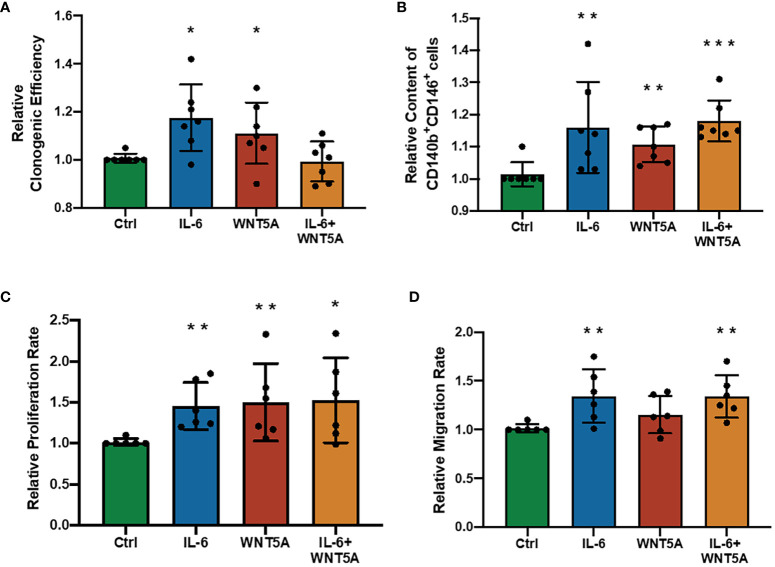
Addition of recombinant human IL-6 and WNT5A on eMSC activities. **(A)** Relative clonogenic efficiency after treatment with GM (green bar), 1,000 pg/ml rhIL-6 (blue bar) or 0.01 μg/ml rhWNT5A (red bar), and a combination of 1,000 pg/ml rhIL-6 and 0.01 μg/ml rhWNT5A (yellow bar) (*n* = 7). **(B)** Relative proportion of CD140b^+^CD146^+^ cells (*n* = 7). **(C)** The relative proliferation rate of eMSC (*n* = 6). **(D)** Relative migration rate of eMSC (*n* = 6). Results are presented as mean ± SD; ^*^ *p* < 0.05; ^**^ *p* < 0.01; ^***^ *p* < 0.001. IL-6, interleukin 6; GM, growth medium; eMSC, endometrial mesenchymal stem cells.

In previous explorations of eMSC regulatory mechanisms, WNT5A was pronounced as a stimulating molecule that can enhance the self-renewal and proliferation of eMSC by activating the WNT/β-catenin signaling pathway (19, 20), which is quite similar to our observation of the regulatory role of IL-6 on eMSC. Thus, we wondered if dual treatment with these two molecules could have a better stimulatory effect on eMSC performances. Treatment of IL-6 or WNT5A alone increased the clonogenic efficiency (*n* = 7, *p* < 0.05, **Figure 5A**), phenotypic expression (*n* = 7, *p* < 0.01, **Figure 5B**), and proliferation rate (*n* = 7, *p* < 0.01, **Figure 5C**) of eMSC efficiently. The migration activity was only enhanced after the IL-6 treatment (*p* < 0.01) but not after the WNT5A alone treatment (*n* = 6, **Figure 5D**). The combined treatment with IL-6 and WNT5A readily increased the phenotypic expression (*p* < 0.001, **Figure 5B**), proliferation activity (*p* < 0.05, **Figure 5C**), and migration activity (*p* < 0.01, **Figure 5D**) of eMSC when compared to the control. However, no significant differences were detected between single and dual treatment groups, suggesting that the addition of IL-6 and WNT5A did not produce an additive or synergistic effect on the endometrial stem cells. Either one of these molecules could trigger the cells to respond.

### Dual treatment of IL-6 and WNT5A enhanced the migrating capacity of eMSC and its therapeutic role in the regeneration of injured mouse endometrium

**Figure 6:**
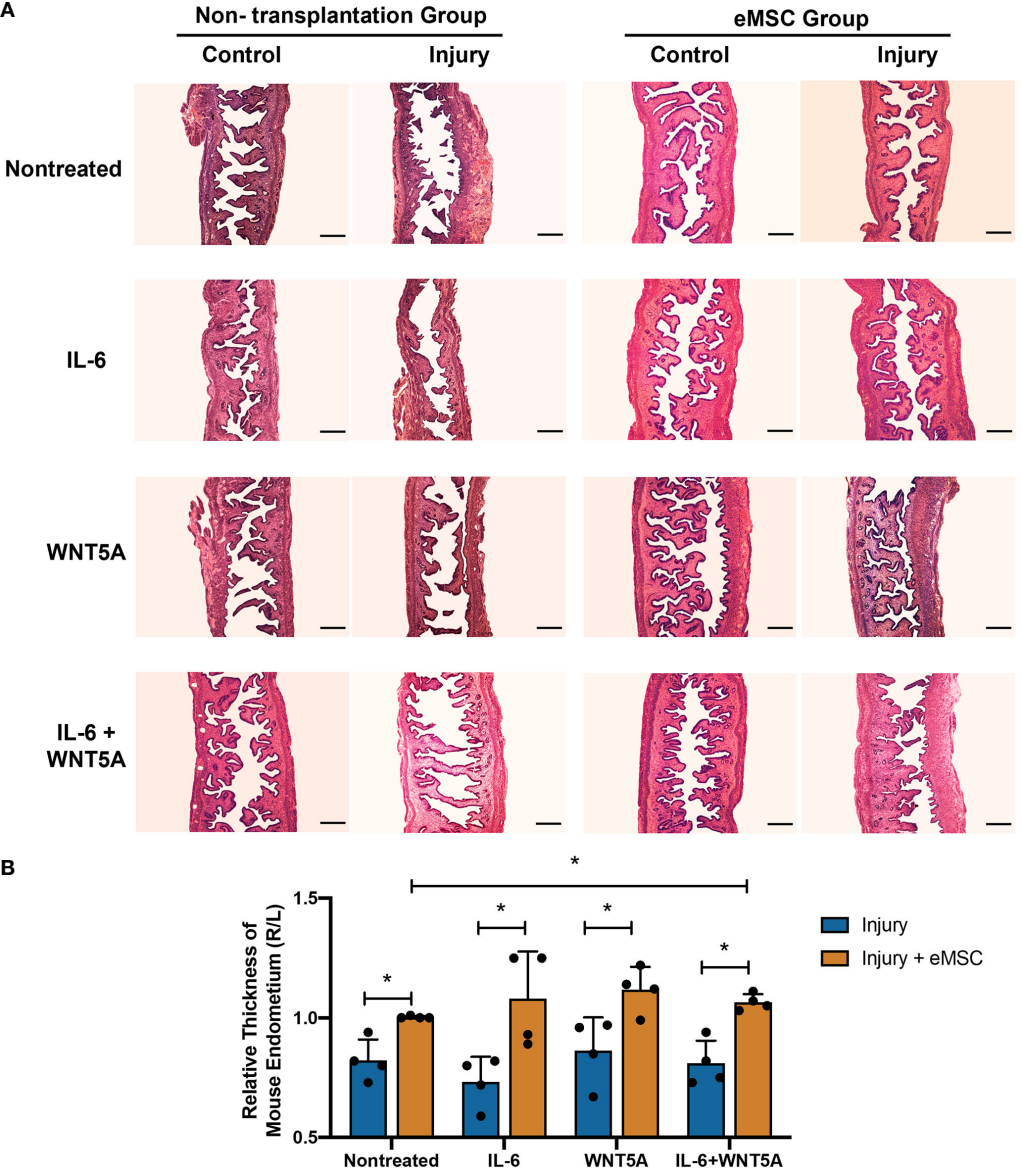
Morphological features of mouse endometrium after transplantation of eMSC under different treatments. **(A)** Representative H&E images of mouse endometrium in different treatment groups. Scale bar: 200 μm. **(B)** Relative thickness of mouse endometrium. Injury group (blue bars) or injury with eMSC group, eMSC pretreated with 1,000 pg/ml rhIL-6 group, eMSC pretreated with 0.01 μg/ml rhWNT5A group, or eMSC pretreated with a combination of 1,000 pg/ml rhIL-6 and 0.01 μg/ml rhWNT5A group (yellow bars) at postoperative day 5 (*n* = 4). The injured uterine horn (right [R]) was normalized against the control uterine horn (left [L]) from the same animal. Results are presented as mean ± SD; ^*^ *p* < 0.05. IL-6, interleukin 6; eMSC, endometrial mesenchymal stem-like cells.

Although *in vitro* dual treatment of IL-6 and WNT5A did not further stimulate the eMSC activities compared to the single treatment groups, the *in vivo* impact of IL-6 and WNT5A on the therapeutic potential of eMSC in endometrial repair was investigated. Utilizing the mouse endometrial injury model, eMSC treated with different niche-related protein(s) were transplanted into the injured uterine horn. All mice that received eMSC with or without treatments displayed better regeneration of the injured endometrium by postoperative day 5 (**Figure 6A**) in terms of increase in tissue thickness when compared to the corresponding treatment condition without eMSC transplantation (*p* < 0.05, *n* = 4, **Figure 6B**). Only eMSC treated with IL-6 and WNT5A displayed better capacity to facilitate repair of the injured endometrium than the nontreated eMSC group. A single treatment of IL-6 or WNT5A alone showed no difference when compared to the nontreated eMSC due to the large sample variation. These findings indicated that the combination of IL-6 and WNT5A might exert a more pronounced effect in stabilizing the performance of eMSC *in vivo*.

**Figure 7:**
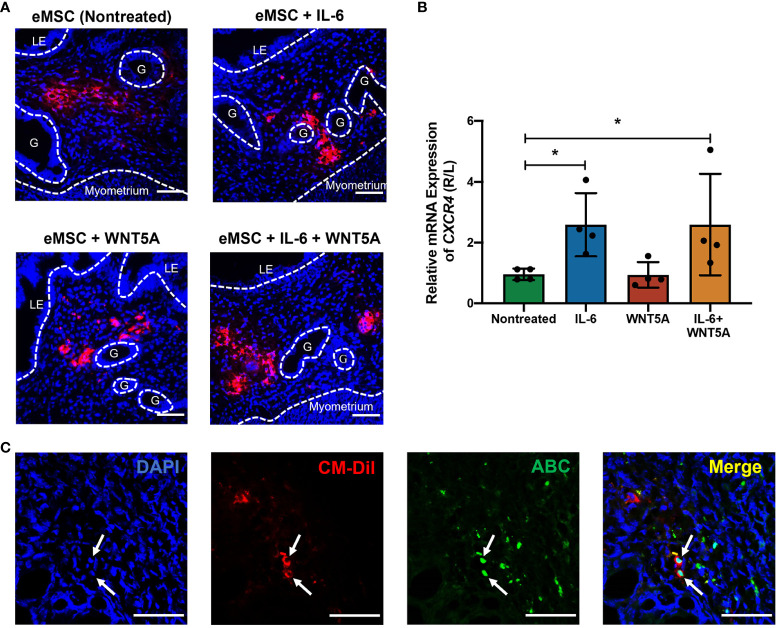
Localization of human eMSC with or without treatment in mouse endometrium and the expression of active β-catenin *in vivo*. **(A)** Representative immunofluorescent images of the localization of human eMSC labeled with CM-Dil (red) in the injured mouse endometrium without and without treatment. Scale bar: 50 μm. **(B)** Relative gene expression of *CXCR4* in eMSC transplantation groups, including nontreated eMSC (green bar), eMSC pretreated with 1,000 pg/ml rhIL-6 (blue bar), eMSC pretreated with 0.01 μg/ml rhWNT5A (red bar), and eMSC pretreated with a combination of 1,000 pg/ml rhIL-6 and 0.01 μg/ml rhWNT5A (yellow bar) (*n* = 4). The injured uterine horn (right [R]) was normalized against the control uterine horn (left [L]) from the same animal. **(C)** Representative immunofluorescent images showing the nuclear expression of ABC (green) in CM-Dil-labeled (red) eMSC pretreated with 1,000 pg/ml rhIL-6 in the injured mouse endometrium. Scale bar: 50 μm. Results are presented as mean ± SD; ^*^ *p* < 0.05. CXCR4; C-X-C motif chemokine receptor 4; LE, luminal epithelium; ABC, active β-catenin; G, glands; eMSC, endometrial mesenchymal stem cells.

The CM-Dil fluorescent dye was used to trace the transplanted eMSC in the mouse endometrium. As shown in **Figure 7A**, the red fluorescence representing CM-DIL labeling was detected in all the eMSC transplantation groups. The CM-DIL dye-labeled eMSC were mainly localized to the upper stromal region of the endometrium for the nontreated and the WNT5A-treated groups. For the other two groups, the fluorescent cells resided at the endometrial-myometrial junction, indicating that eMSC with IL-6 treatment might have greater migration capacity in the engrafted endometrium. Quantitative analysis of the migration indicator *CXCR4* revealed that single treatment of IL-6 and combined treatment (*n* = 4, *p* < 0.05, **Figure 7B**) significantly increased the migration capacity of eMSC *in vivo*, which was consistent with the *in vitro* results. Furthermore, nuclear expression of ABC was detected in eMSC with IL-6 treatment alone (white arrows, **Figure 7C**). These results, together with the *in vitro* functional assays, demonstrated that exogenous IL-6 treatment can activate the WNT/β-catenin signaling in eMSC.

## Discussion

Menstruation involves a complex series of inflammatory events, resulting in tissue breakdown and bleeding (23, 24). The influx of inflammatory cells resulting from progesterone withdrawal and the interaction between these immune cells and decidualized stromal cells can mediate the release of a wide spectrum of proinflammatory factors (25). These cytokines from the inflammatory milieu have been reported to influence MSC functionalities by inducing cell proliferation, lodging, and migration, as well as controlling the fate between quiescence and activation of primitive stem cells (26, 27). This phenomenon was also observed in eMSC when cocultured with endometrial cells from the menstruation phase, and IL-6 was identified as a potential modulator during this period (16). To gain a better understanding of IL-6 functions and its production in the uterine environment, we examined the expression and secretion of IL-6 in endometrial cells across menstrual cycles. Also, we confirmed the responsiveness of our eMSC to the surrounding IL-6 cytokine. Both *in vitro* and *in vivo* functional assays demonstrated a positive impact of IL-6 on the maintenance of eMSC, and WNT/β-catenin signaling was found involved during this regulatory process. To the best of our knowledge, this is the first study focusing on the role of IL-6 and its interaction with WNT/β-catenin signaling during the regulation of endometrial mesenchymal stem-like cells.

The production of IL-6 was reported to be negatively correlated with the concentration of progesterone in human gingival fibroblasts (28). However, the secretion of IL-6 across the menstrual cycle remains controversial. Some studies reported that the plasma concentration of IL-6 reaches its lowest during the luteal phase and increases sharply in the preovulatory phase, displaying an opposite trend when compared to the progesterone level (29, 30). However, other studies showed that the plasma level of IL-6 remained unchanged throughout the menstrual cycle (31). A more recent study reported that the IL-6 level was distinctly higher in the menstrual blood than in the plasma (2), indicating that the physiological changes during menstruation can regulate the endogenous production of IL-6 in the human endometrium. Previously, our group reported an elevation of the IL-6 level in the eMSC spent medium after coculture with menstruation-phase endometrial cells, providing evidence that endometrial cells might be one of the sources of endogenous IL-6 (16). In the present study, the level of *IL-6* mRNA in endometrial stromal cells was found to be higher during menstruation and the proliferative phase than the secretory phase, which coincided with the changes in progesterone level across the menstrual cycle. Also, hypoxia has been reported to induce the production of IL-6 in many somatic cell types such as hepatocytes, fibroblast-like synoviocytes, and cardiac fibroblasts (32–34). A similar phenomenon was also observed in endometrial cells. Endometrial shedding during menstruation leads to a prominent decline in oxygen content, thus creating a hypoxic condition to the resident cells. The IL-6 released from endometrial cells in hypoxia environment was significantly more than that from normoxia condition, indicating that the oxygen level of the microenvironment can influence the secretion of IL-6.

IL-6 has been proven to exhibit pro- and anti-inflammatory properties and affect the biological activities of various cell types, including the MSC population (35). In fact, MSC acts as both the source and target of IL-6 (36). They can exert their immunomodulatory effects through cytokine secretion containing IL-6 (37). However, the IL-6 content in the spent medium of eMSC was relatively low (16), suggesting that the eMSC may not be the dominant source of endogenous IL-6. Since IL-6 exhibited its biological effects through binding to the IL-6R in the target cells (38), the expression of IL-6R in our eMSC population was further confirmed in the present study. Cells with coexpression of stem cell markers (CD140b and CD146) and IL-6R were found to reside in the perivascular region of the human endometrium, indicating the capacity of eMSC to respond to the endogenous IL-6. Also, quantification of the IL-6R in the purified eMSC population showed that about half of the eMSC population expresses IL-6R, providing the possibility that exogenous IL-6 can alter the fate of eMSC.

In previous studies, IL-6 was reported as a potential “stemness” gene of MSC (36). The expression of IL-6 remained high in the undifferentiated state of MSC and declined dramatically after osteogenic differentiation. Also, treatment with IL-6 was proven to stimulate the cell proliferation of MSC (39, 40). However, the action of exogenous IL-6 on MSC maintenance is conflicting (41, 42). Our *in vitro* assays demonstrated that the addition of recombinant IL-6 protein promoted the proliferation, phenotypic expression, and colony-forming capacity of eMSC. Together, these findings suggest that the abundance of IL-6 during endometrial breakdown is likely to contribute to the activation of eMSC during menstruation. In another aspect, IL-6 was reported to stimulate the migration and invasion of trophoblast (43). Thus, it is not surprising that this cytokine is also involved in the migration of MSC (44). Specifically, using a 3D steroid culture system, Casson et al. demonstrated that the MSC migrated toward the IL-6 signal in both 2D and 3D cultures (45). In the present *in vivo* and *in vitro* experiments, the migration capacity of human eMSC was significantly elevated upon stimulation by exogenous IL-6. The IL-6-treated eMSC expressed more *CXCR4*, a chemokine receptor involved in the migration and movement of cells (46). By posttransplantation day 5, most of the IL-6-pretreated human eMSC resided in the lower layer of the endometrium near the endometrial–myometrial junction. While the nontreated eMSCs were mainly localized to the stromal region around the luminal epithelium, it is worth noting that several gynecological pathologies result in abnormally high levels of IL-6. For instance, high concentration of IL-6 is observed in the peritoneal fluid of women with endometriosis (47, 48). The exposure of IL-6 may inflict the stem cells that shed along with retrograde menstrual effluent to migrate, invade, and establish endometriosis at ectopic sites.

Accumulating evidence shows that IL-6 can interact with WNT/β-catenin signaling. For example, hypoxia-induced IL-6 secretion can activate WNT signaling in rat MSC through the JAK2/STAT3 signaling (49). Yoshida et al. demonstrated that IL-6 activated the WNT/β-catenin signaling in fibroblast-like synoviocytes by reducing the production of DKK1, a well-known WNT antagonist (50). In another study, IL-6 had inhibitory effects on WNT activation in osteoblasts (51). In this study, the expression of active β-catenin and the level of the TCF/LEF transcriptional activities in eMSC increased after treatment with recombinant IL-6 protein, indicating the activation of WNT/β-catenin signaling. The addition of WNT inhibitor IWP-2 abolished the stimulatory effect of rhIL-6 on cell proliferation, colony-forming capacity, and phenotypic marker maintenance of eMSC. The elevation in the expression of active β-catenin was also suppressed after the addition of IWP-2 to the culture system. The *in vivo* assay confirmed that eMSC pretreated with IL-6 possessed the expression of active β-catenin at day 5 postoperation. These results suggested that IL-6 from endometrial cells at menstruation activated WNT and promoted the functional and phenotypic properties of eMSC. Since both WNT5A and IL-6 positively regulate the eMSC cell fate by activating WNT/β-catenin signaling, the effect of dual treatment of these two molecules was investigated. Combining IL-6 with WNT5A resulted in an increase in the phenotypic expression, proliferation, and migration activity of eMSC. These properties were further demonstrated after the transplantation of eMSC treated with both these molecules, resulting in better restoration of the injured mouse endometrium. The use of complementary RNA sequencing will be helpful to better understand the underlying mechanisms of the observed stimulatory effect in future studies.

## Conclusion

We demonstrated in this study that endometrial cells during menstruation produced high amounts of IL-6 in the microenvironment. The presence of IL-6 induced the stem cells in the human endometrium to proliferate and migrate through activation of the WNT/β-catenin pathway. Treatment of eMSC with IL-6 or WNT5A enhanced their therapeutic potential in the regeneration of injured endometrium, providing new insights for eMSC activation to enhance the efficiency of cell-based therapy.

## Data availability statement

The original contributions presented in the study are included in the article/**Supplementary Material**. Further inquiries can be directed to the corresponding author.

## Ethics statement

The studies involving humans were approved by Institutional Review Board of the University of Hong Kong/Hospital Authority Hong Kong West Cluster and the Institutional Review Board of the University of Hong Kong-Shenzhen Hospital. The studies were conducted in accordance with the local legislation and institutional requirements. The participants provided their written informed consent to participate in this study. The animal study was approved by Committee of Use of Live Animals in Teaching and Research, The University of Hong Kong. The study was conducted in accordance with the local legislation and institutional requirements.

## Author contributions

TL: Data curation, Formal analysis, Investigation, Methodology, Validation, Writing – original draft, Writing – review & editing. HL: Methodology, Resources, Writing – review & editing. EN: Funding acquisition, Methodology, Resources, Writing – review & editing. WY: Conceptualization, Funding acquisition, Supervision, Visualization, Writing – review & editing. PC: Conceptualization, Supervision, Visualization, Writing – review & editing. RC: Conceptualization, Funding acquisition, Methodology, Supervision, Writing – original draft, Writing – review & editing.

## Supplementary material

Supplementary Figure 1 Flow cytometry for IL-6R^+^ CD140b^+^ CD146^+^ cells. **(A)** The top panel outlines the gating strategy to obtain single cells for analysis. **(B)** The bottom panel are representative images for each fluorochrome for analysis of IL-6R^+^ CD140b^+^ CD146^+^ cells.

Supplementary Figure 2 Uncropped scan of western blots (). The representative uncropped western blotting images is highlighted with red squares. The protein levels of ABC in eMSC in GM (band 1), 1000 pg/ml of rhIL-6 (band 2), IWP-2 at 1.25 μM (band 3) and 1000 pg/ml of rhIL-6 with IWP-2 at 1.25 μM (band 4).
