# Supplementary figures and images for: Interleukin 6 at menstruation promotes the proliferation and self-renewal of endometrial mesenchymal stromal/stem cells through the WNT/β-catenin signaling pathway
Source: Front Immunol. 2024 May 3;15:1378863. doi: 10.3389/fimmu.2024.1378863 (PMC11099287; doi:10.3389/fimmu.2024.1378863)

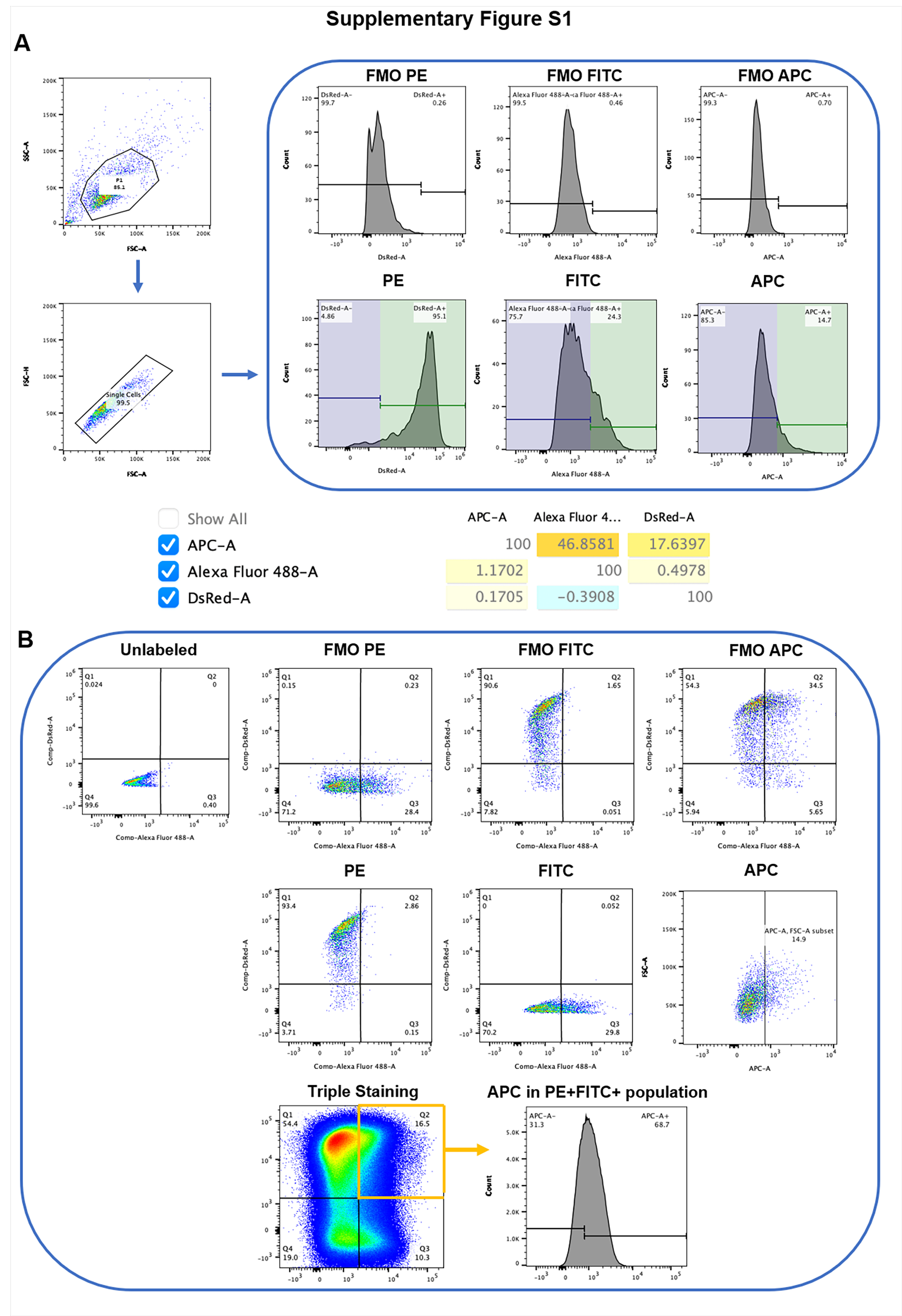

Supplement: Supplementary Figure 1 — Flow cytometry for IL-6R+ CD140b+ CD146+ cells. (A) The top panel outlines the gating strategy to obtain single cells for analysis. (B) The bottom panel are representative images for each fluorochrome for analysis of IL-6R+ CD140b+ CD146+ cells. [file Image_1.tif]

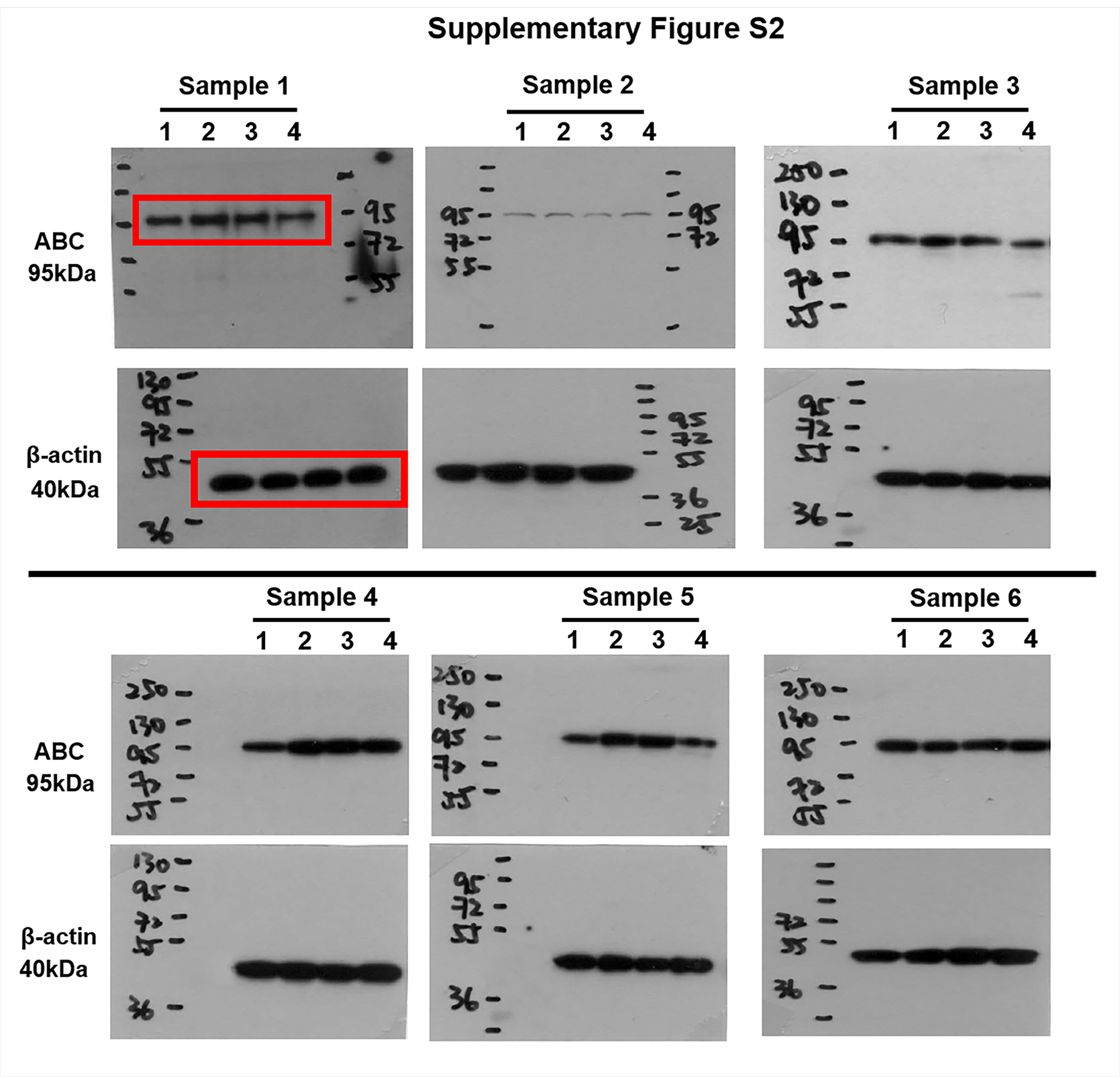

Supplement: Supplementary Figure 2 — Uncropped scan of western blots (). The representative uncropped western blotting images is highlighted with red squares. The protein levels of ABC in eMSC in GM (band 1), 1000 pg/ml of rhIL-6 (band 2), IWP-2 at 1.25 μM (band 3) and 1000 pg/ml of rhIL-6 with IWP-2 at 1.25 μM (band 4). [file Image_2.tif]
