# Supplementary material for: Interleukin 6 at menstruation promotes the proliferation and self-renewal of endometrial mesenchymal stromal/stem cells through the WNT/β-catenin signaling pathway
Source: Front Immunol. 2024 May 3;15:1378863. doi: 10.3389/fimmu.2024.1378863 (PMC11099287; doi:10.3389/fimmu.2024.1378863)
Supplement: Supplementary file 3 [file DataSheet_1.docx]

**Supplementary Table S1 – Pathological characteristic of full thickness endometrial samples**

| **Patient No.** | **Age** | **Menstrual Phase** | **Diagnosis** |
| --- | --- | --- | --- |
| 1 | 44 | Secretory | Adenomyosis + Leiomyomas |
| 2 | 47 | Secretory | Leiomyomas |
| 3 | 49 | Secretory | Leiomyomas |
| 4 | 49 | Secretory | Adenomyosis |
| 5 | 46 | Secretory | Leiomyomas |
| 6 | 46 | Secretory | Leiomyomas |
| 7 | 50 | Secretory | Adenomyosis + Leiomyomas |
| 8 | 49 | Secretory | Adenomyosis + Leiomyomas |
| 9 | 45 | Secretory | Leiomyomas |
| 10 | 47 | Secretory | Leiomyomas |
| 11 | 44 | Proliferative | Leiomyomas |
| 12 | 41 | Proliferative | Leiomyomas |
| 13 | 44 | Proliferative | Leiomyomas |
| 14 | 46 | Proliferative | Adenomyosis |
| 15 | 43 | Proliferative | Adenomyosis |
| 16 | 46 | Proliferative | Leiomyomas |
| 17 | 46 | Proliferative | Adenomyosis |
| 18 | 48 | Proliferative | Leiomyomas |
| 19 | 48 | Proliferative | Adenomyosis |
| 20 | 47 | Proliferative | Leiomyomas |
| 21 | 44 | Proliferative | Leiomyomas |
| 22 | 47 | Proliferative | Leiomyomas |
| 23 | 50 | Proliferative | Leiomyomas |
| 24 | 45 | Proliferative | Leiomyomas |
| 25 | 44 | Proliferative | Adenomyosis + Leiomyomas |
| 26 | 48 | Proliferative | Leiomyomas |
| 27 | 43 | Proliferative | Leiomyomas |

**Supplementary Table S2 – Age of menstruation samples**

| **Patient No.** | **Age** | **Menstrual phase** |
| --- | --- | --- |
| 1 | 33 | Menstrual |
| 2 | 37 | Menstrual |
| 3 | 34 | Menstrual |
| 4 | 35 | Menstrual |
| 5 | 32 | Menstrual |
| 6 | 40 | Menstrual |

**Supplementary Table S3 – Primary and secondary antibodies used for immunofluorescent staining**

| **Primary antibody** | **Dilution** | **Company** | **Catalog number** |
| --- | --- | --- | --- |
| Goat polyclonal PDGFRβ | 1:100 | R&D Systems | AF385  (RRID: AB_355339) |
| Mouse polyclonal CD146 | 1:100 | Novus | NBP1-88132  (RRID: AB_11008437) |
| Rabbit polyclonal IL-6R | 1:100 | Biorbyt | orb228601  (RRID: AB_3083788) |
| Mouse monoclonal active β-catenin | 1:100 | Millipore | 05-665  (RRID: AB_309887) |
|  |  |  |  |
| **Secondary Antibody** | | **Dilution** | **Company** |
| Donkey anti-mouse IgG Alexa 488 | | 1:200 | Life technologies |
| Donkey anti-rabbit IgG Alexa 568 | |  |  |
| Rabbit anti-goat IgG Alexa 647 | |  |  |
